# Midwifery in London
**Report on the Provision of Midwifery Service in the County of London*. By Janet Lane-Claypon, M.D., D.Sc. (L.G.B. Reports.)


**Published:** 1917-12-08

**Authors:** 


### MIDWIFERY IN LONDON.*

By A SOCIAL WORKER.

The information gathered in Dr. Janet Lane-Claypon's report should be of use to many workers. We could wish to see a booklet made from her account of general conditions, detached from statistics needed by the L.G.B., and widely circulated. The figures given, compiled as they are from statistics of the Registrar-General, of M.O.H.s, and of institutions, represent immense labour, and point to the advisability of a co-ordinated system. They also reveal some curious facts. Thus while 101,649 births occurred among residents in the County of London, 97,451 notifications only were received, and the deficit exceeds this number if the births to non-residents in the county are added. The variation, again, in numbers attended from institutions is remarkable, Stepney showing 40.5 per cent., and Poplar 11.2 per cent. The relative poverty of the two districts is not such as to account for this difference. Either, therefore, the habit of seeking free assistance is more common, or the number of medical students or pupil-midwives training in the district is such as to make attendance necessary. Since a minimum of twenty births is generally required to qualify men or women students, Dr. Lane-Claypon is of opinion that the 7,000 cases (approximately) attended by them in 1915 exactly met the actual need; so that attendance was practically only given for this purpose. Pupil-midwives, on the other hand, do not actually take charge of cases, and many of them qualify on births occurring within institutions. Many of the Samaritan funds of hospitals for mothers and babes arose from the misery seen, and in some sense endured, by students attending out-patients. But so long as ante-natal visitation of homes or examination of the mother are not customary there can be no assurance that these or any other form of maternity charities will reach the persons most in need. Considering the serious danger both to mother and child in cases where extreme poverty or temporary misfortune or unthrift cause the maternity benefit to be diverted to general purposes, and, indeed, certain cases in which it is not received at all, this is to be regretted. Even the maternity bag, as the report points out, may fail in its useful purpose if the loan results in no clothes being provided for the baby to wear after the return of the bag, or if from want of proper care it carries on septic infection.

Yet another form of charity is shown to need careful reconsideration and reorganisation. The arrangement made by some old-established charitable institutions with private midwives, which gives a fee of only 6s. a case, plus a small sum for appliances, or a supply of these, is long out of date. As Dr. Lane-Claypon caustically remarks, the charity appears to be mainly on the part of the midwife; and the chief reason why any qualified woman is willing to accept such cases is probably that the common worry of the doctor's fee does not arise, the institution sending its own medical officer or paying a practitioner, so that the cases may usefully be taken to help in training pupils.

Roughly speaking, the saving of time to a midwife by pupils is estimated at about one-third, and it is practically only by such help that she can hope to make her practice pay, even if work is carried to the limit of human strength. The difficulties and hardships of the midwife's life have been freely discussed of late, but there seems no doubt that much remains to be said and done before this most important service to the State can be established on a proper basis. For instance, the development of the work of maternity centres and clinics shows how necessary the rising standard of opinion makes it that the midwife should have more time for ante-natal attention. At present she may suspect or perceive need for medical treatment, and suggest to her patient to obtain it at a centre. Dr. Lane-Claypon estimates that this would be desirable in some 40 per cent. of cases. But many women will be found nervous and reluctant to go alone. Very probably a midwife has been engaged in decided preference to a doctor, and if the matter is pressed the patient may transfer herself to another midwife. If the woman who advised the treatment could go with her, much would be gained. Many midwives would willingly give more time to this and other ante-natal care if better pay enabled them to take fewer cases, and some do so, especially those who have pupils. Some, indeed, have considerably raised their fees, in one case known to the present writer the charge of 10s. eighteen months ago is now 17s. But the increased cost of living and of appliances will swallow most of the gain. The maternity benefit unfortunately acts as a positive hindrance to ante-natal attention by largely doing away with the plan by which the mother took round small sums as she could afford them in payment of the impending fee. Opportunity for inquiry and advice thus came naturally. Meantime pupil-midwives are not as numerous as they should be, and many who qualify do not intend to practise. Expenses are heavy, bad debts have to be reckoned with, and the vexatious question of the doctor's fee in abnormal cases urgently needs solution if the profession is to attract desirable women. Dr. Lane-Claypon pays a pleasant tribute to the kindly assistance of doctors generally to the midwife. But the raised standard of actual maternity nursing desired from her must increasingly add to the strain of her work.

Many readers of the report will be surprised by the number of confinements attended in the homes by doctors. Actually 80 per cent. of all births are found to be in classes where health visitors can enter. Doctors attended 40,000 births in the year, and Dr. Lane-Claypon is probably not far wrong in calculating that 20,000 of these were among people below the standard of the well-to-do! This figure brings up the very important question of the *handy-woman*. We may hope that the practice of "covering," when some understanding exists that the doctor shall not be sent for unless or until there is serious need, is not common. But all experience goes to show that while the midwife is fetched at once, all concerned are unwilling to waste a doctor's time during early stages. Partly for this reason, but also partly because some experience of normal cases is apt to convince the handy-woman that she can quite well manage an ordinary confinement herself, she is apt to trust her own ability. In any case serious responsibility for the incidentals of maternity nursing rests upon her, whatever her standard of care or cleanliness; she may be taking the entire charge of house and children for the time, perhaps doing the washing also. Her wages, too, are rising, and now are apt to involve a demand for food or beer, or both, but they vary very greatly with the standard of the

* *Report on the Provision of Midwifery Service in the County of London.* By Janet Lane-Claypon, M.D., D.Sc. (L.G.B. Reports.)

street, and the skill and attention expected. The report seems to assume that such a woman is generally engaged, but in a great many cases, especially when a midwife is employed, a relative or "the lady downstairs" performs the service Hence the growing need for a general teaching of hygiene. Some encouraging results already follow the efforts made, as, for instance, the widespread conviction that "there isn't not a minute to lose" when symptoms of ophthalmia neonatorum appear. Recommendations for the instruction of women undertaking care when no midwife is employed have been made in several quarters. It is certainly needed, and also in the poorest classes, especially in the case of young mothers, friendly personal visiting and advice as to preparation would often be helpful. Clothing for the baby is not always provided in time. Dr. Lane-Claypon suggests that for mothers who cannot sew much, good second-hand clothing may be cheaply bought and well boiled, and is better than cheap new things. Bedding in an unfit state may be treated by the sanitary authority.

With regard to in-patient accommodation, Dr. Lane-Claypon believes it to be sufficient in the Metropolitan area, but unequally distributed. As at present used, of normal and abnormal cases received, a proportion varying from 32 to 66 per cent. were primiparous women; in the case of the higher figure 50 per cent. were unmarried. Complicated cases averaged 9 per cent. A useful appendix gives particulars of the maternity work of the twelve medical schools, and of other institutions providing midwifery service.

The "Memorandum on the Present and Future Position of the Midwife," presented by Dr. E. W. Hope at the annual meeting of the Association for Promoting the Training and Supply of Midwives, sums up the perplexing conditions, and recommends a subsidised training and subsidised service on the analogy of Indian Forestry and other Government careers, and of midwifery itself as carried out in Denmark, for example. Dr. Hope expects after the war a large increase in the birth-rate, and points to the wastage of qualified doctors in the war, and of medical students by the calling up of men below four years of study. He suggests that the midwife better than anyone else can hope so to deal with sequelæ of venereal disease that healthy children shall replace stillbirths, and he argues that the worry of doctors' fees should be removed from her by a plan similar to the expedient used at Liverpool, whereby the City Council assures the fee, part is recovered, if possible, from the patient, and part paid by the L.G.B. Some £80,000 to £100,000, he estimates, would be required for subsidies. We are growing accustomed to the prospect of expending larger sums than this on social services! But Dr. Hope's statement that of 40,000 women registered, not half are practising, brings home the seriousness of the question.

